# Supplementary figures and images for: Usefulness of Sepsis-3 in diagnosing and predicting mortality of ventilator-associated lower respiratory tract infections
Source: PLoS One. 2021 Jan 14;16(1):e0245552. doi: 10.1371/journal.pone.0245552 (PMC7808583; doi:10.1371/journal.pone.0245552)

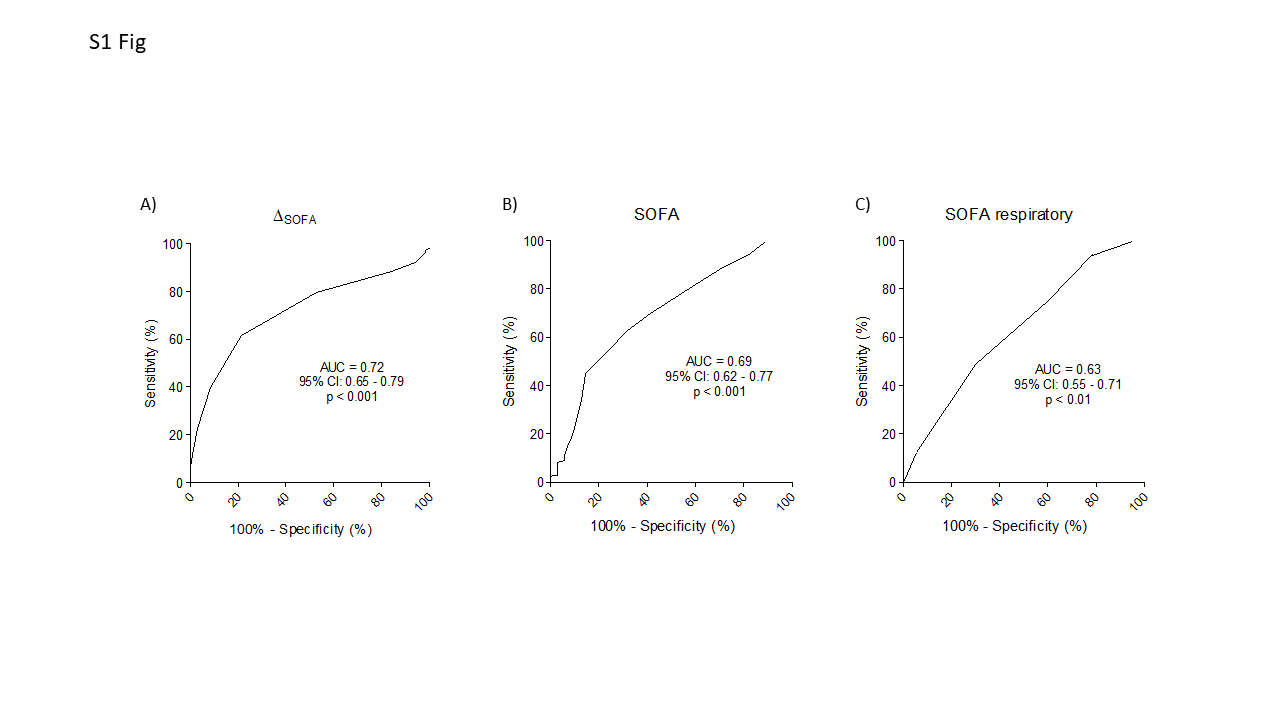

Supplement: S1 Fig — SOFA sequential organ failure assessment. (TIF) [file pone.0245552.s001.tif]

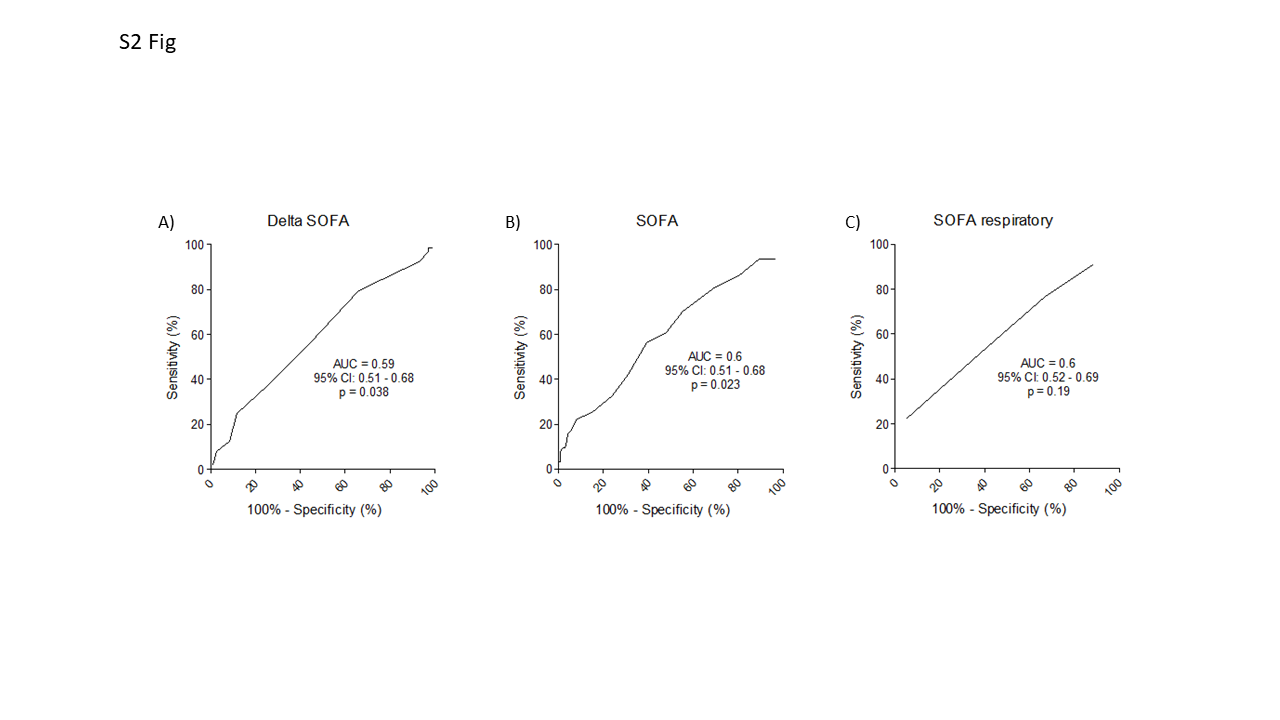

Supplement: S2 Fig — SOFA sequential organ failure assessment. (TIF) [file pone.0245552.s002.tif]
