# Supplementary material for: Usefulness of Sepsis-3 in diagnosing and predicting mortality of ventilator-associated lower respiratory tract infections
Source: PLoS One. 2021 Jan 14;16(1):e0245552. doi: 10.1371/journal.pone.0245552 (PMC7808583; doi:10.1371/journal.pone.0245552)
Supplement: S1 Appendix — (DOCX) [file pone.0245552.s003.docx]

S1 Appendix. Criteria for diagnosis of VAT and VAP

|  | **VAT** | **VAP** |
| --- | --- | --- |
| **Clinical** | At least 2 of:   - Body temperature > 38.5°C or < 36.5°C - Leukocyte count > 12,000 cells/µL or < 4,000 cells/µL - Purulent endotracheal aspirate | |
| **Microbiology** | Positive culture of potentially pathogenic microorganism on endotracheal aspirate of ≥ 10^5^ CFU/mL, or bronchoalveolar lavage of 10^4^ ≥ CFU/mL | |
| **Chest X-ray** | Absence of new or progressive pulmonary infiltrates | Presence of new or progressive pulmonary infiltrates |
