# Supplementary material for: Usefulness of Sepsis-3 in diagnosing and predicting mortality of ventilator-associated lower respiratory tract infections
Source: PLoS One. 2021 Jan 14;16(1):e0245552. doi: 10.1371/journal.pone.0245552 (PMC7808583; doi:10.1371/journal.pone.0245552)
Supplement: S2 Appendix — (DOCX) [file pone.0245552.s004.docx]

S2 Appendix. Sepsis-3 criteria for diagnosis of sepsis and septic shock

| **Sepsis** | Both following criteria must be reached:   - Acute change in total SOFA score ≥ 2 points - Consequent to an infection |
| --- | --- |
| **Septic shock** | Subset of sepsis, reaching following criteria:   - Persisting hypotension requiring vasopressors to maintain MAP ≥ 65 mmHg - Serum lactate level > 2 mmol/L (18 mg/dL) - Despite adequate volume resuscitation |
