# Supplementary material for: Usefulness of Sepsis-3 in diagnosing and predicting mortality of ventilator-associated lower respiratory tract infections
Source: PLoS One. 2021 Jan 14;16(1):e0245552. doi: 10.1371/journal.pone.0245552 (PMC7808583; doi:10.1371/journal.pone.0245552)
Supplement: Attachment — Submitted filename: Response to Reviewers.docx [file pone.0245552.s005.docx]

Dear Editors,

Dear Reviewers,

Thank you for your helpful suggestions regarding our manuscript.

Following your comments, we have provided some additional information which, we believe, should improve the quality of the article.

Please find appended below in italic our point-by-point response to your questions:

**Reviewer #1:** This retrospective study evaluated diagnostic ability of sepsis-3 criteria, total SOFA and respiratory SOFA to differentiate VAP from VAT, and concluded that sepsis-3 was not useful. It is worth publishing, since logic looks appropriate and conclusion is reasonable. However, it also includes several problems to be addressed.

1. P6, L90 “All adults patients with a diagnosis of VA-LRTI were retrospectively included in the study. The diagnosis of VA-LRTI was prospectively performed by ICU physicians.” If authors regarded diagnosis was made prospectively, they had to review not only patients diagnosed as VA-LRTI, but also all patients admitted to the ICU during study period. If they had reviewed only retrospectively-included VALRTI patients, prospective diagnosis of VALRTI could not be possible. Or did they only diagnose VAT or VAP among VALRTI patients? Please explain and describe clearly in the Methods section.

*Thank you for pointing out this lack of clarity in our initial formulation. Physicians in charge of the patients identified VA-LRTI episodes as they occurred (prospective diagnosis), and retrospectively collected data on these patients. From a methodological point of view, this is therefore a retrospective study. Accordingly, and to avoid confusion, we have changed the description of the study design in the methods section into: “**Continuous surveillance of ICU-acquired infections allowed prospective identification of patients with VA-LRTI. These patients were subsequently included in this retrospective study and other data were extracted from electronic files”.*

2. P7, L120 Regarding Objective of this study, “accuracy of predicting ICU mortality” was evaluated only in sepsis-3 analysis, not in analyses of total SOFA and respiratory SOFA. However, since authors repeatedly compared sepsis-3 to SOFA in the context of this study, they should have evaluated SOFA’s ability in predicting mortality. Otherwise, they should focus only on ability of diagnosis of VAT and VAP in comparison between sepsis-3 and SOFA, and eliminate results of mortality in spsis-3 analysis.

*We fully agree with your comment, and have therefore completed the manuscript by reporting the assessment of total SOFA and respiratory SOFA in predicting mortality in main text, Table 5, Figures 5 and 6, and Figure S2.*

**Reviewer #2:**

1. The definition was vague. Did the authors look for signs of pulmonary consolidation in admission? no CT scan?

*Thank you for pointing out these important matters. To avoid any confusion for the reader, we now explicitly mention in the manuscript that criteria from the International ERS/ESICM/ESCMID/ALAT guidelines for the management of hospital-acquired pneumonia and ventilator-associated pneumonia were used for the definition of VAT and VAP*^1^*. Furthermore, these criteria are now described in S1 Appendix, in addition to criteria detailed in the methods section.*

*This definition does not specify whether chest X-ray or CT-scan should be used for the diagnosis of VA-LRTI. In our study, only chest X-ray was used to distinguish VAT from VAP as CT-scan is not routinely performed for the diagnosis of VAP, due to greater cost, increased time required to obtain images, higher radiation exposure and risks associated with intra-hospital transportation*^2^*.*

*We have therefore completed the methods section of our manuscript, by explicitly stating that CT-scan was not used to differentiate VAT from VAP in our study.*

*We acknowledge that* *lung opacities were probably missed in some patients with VAP, given the reported lack of sensitivity of chest X-ray for the detection of pneumonia*^3,4^*.* *However, the use of chest X-ray allowed us to diagnose VAT and VAP using the same criteria than in the TAVeM study. Accordingly, our definitions of VAP and VAT are consistent with those in which a difference in mortality rates were reported*^5^*.*

*These points are now mentioned in the limitations section of the manuscript.*

2. What is category of admission? Please explain details.

*We acknowledge that clarifications were needed regarding definitions of admission categories.*

*We are actually referring to the type of admission in ICU.* *Admission were defined as surgical if consecutive to a surgery, and medical in the opposite case.*

*The term “Category of admission” has been replaced by “Admission type”, and these definitions are now specified in caption of Table 1.*

3. The adjusting the severity is important.

*We thank you for raising the important question of adjusting on severity in our analysis.*

*As pointed out in your comment, total SOFA at ICU admission was significantly higher in VAP than in VAT. Assessment of SAPS2 at ICU admission also showed a trend for higher severity in VAP.*

*We perfectly acknowledge that confounding factors such as higher levels of severity at ICU admission may partly explain further increases in ∆SOFA in patients with VAP. Addressing this issue would be crucial from the prospect of a study aiming to understand the mechanisms associated with the development of VAP.*

*However, we’d like to remind that the goal of our study was merely diagnostic. Accordingly, the practical question that our study aimed to answer is whether a worsening in organ failures could be used as a reliable criterion to differentiate VAT from VAP, and could therefore be used in the daily clinical practice to decide whether to initiate or not antibiotic treatments. To answer this practical question, it appears that severity criteria, including ∆SOFA, must be assessed as they would by the physician at the patient’s bedside, without adjustment for potential confounders.*

*Initial version of our manuscript lacked clarity regarding these points, which have now been developed in the discussion section of the revised version. We believe it will help the reader to understand more clearly the purpose of our study.*

4. From reading the paper many things remain unclear. What is special about this study in relation to future strategy or research? The study lacks clarity and furthermore, it does not become clear what is particulary special and what the readers can learn from it.

*The rationale of our study mainly relies on the 3 following points:*

*a) Early initiation of antibiotic treatment is recommended in VAP, while there is currently no recommendation regarding such a strategy in VAT*^6^*. Therefore, finding reliable criteria for the early distinction between VAT and VAP seems relevant.*

*b) Physicians may experience difficulties in making the early distinction between VAT and VAP in the daily clinical practice, due to confounding factors on chest X-ray images (pleural effusion, lung edema)*^3,4^ *or because of the frequent delay in appearance of lung opacities*^7^*.*

*c) Data from the TAVeM study show that patients with VAP experience greater severity of illness than those with VAT, thus suggesting that a diagnosis of sepsis, reflecting a worsening in organ failures, could be found more frequently in VAP compared to VAT.*

*Therefore, our study aimed to clarify a practical question for the daily clinical practice, i.e. whether diagnosis of sepsis according to Sepsis-3 criteria, reflecting a greater severity of illness, would be a reliable criterion to diagnose VAP in patients with VA-LRTI, and accordingly, drive the early initiation of antibiotic treatment.*

*Our results suggest that assessment of severity, especially through Sepsis-3 criteria, exhibit poor performances to early differentiate VAT from VAP. Importantly, we found poor sensitivity and negative predictive value of these criteria to rule out a diagnosis of VAP. As a consequence, these criteria should probably not be used to drive the early initiation of antimicrobial therapy in patients with evidence of VA-LRTI. Especially, the absence of diagnosis of sepsis appears as a bad criterion to decide not to initiate antibiotic treatments.*

*To the best of our knowledge, our study is the first to address this question, and we believe it could have a practical impact for the physician at the patient’s bedside.*

*However, we acknowledge that our message was not enough clearly developed in our manuscript. Accordingly, these points are now clearly developed in the first part of our discussion. We hope these changes will help bringing more clearly the aims and results of our study to the reader.*

**Reviewer #3:** I greatly appreciate giving me the opportunity to review the manuscript entitled “Usefulness of Sepsis-3 in diagnosing and predicting mortality of ventilator-associated lower respiratory tract infections”. It is true that the more accurate and rapid diagnostic tool to distinguish VAP and VAT is necessary to improve the management of critically ill ventilated patients. However, the aim of the current study is not clear, and the study design seems not optimal. My concerns are as follows.

1. First, the use of the term “sepsis-3 criteria” is not appropriate. Sepsis-3 is just “Sepsis-3, i.e., it is used to define sepsis based on progress of organ dysfunctions, and it is not a general diagnostic tool for other diseases. Therefore, the authors must use the term such as “increase in SOFA score”, not “sepsis-3 criteria”.

*We acknowledge that the term “Sepsis-3 criteria” was not used in an appropriate way in our manuscript. Especially, use of this term may be confusing as it does not indicate whether criteria for sepsis or septic shock were evaluated. However, the main purpose of our study was to assess whether the presence of sepsis would be a good marker of VAP, and conversely, if absence of sepsis would be sufficient to rule out VAP, and therefore decide not to initiate antibiotic treatment. As a consequence, we believe it is important for a matter of clarity to keep reminding to the reader that our study evaluated the diagnostic criteria for sepsis.*

*Therefore, we have replaced the term “Sepsis-3 criteria” by “sepsis criteria according to the Sepsis-3 definition” throughout the article. Furthermore, description of criteria for sepsis and septic shock according to the Sepsis-3 definition has been added in S2 Appendix.*

2. The aim of the study is unclear. If the authors have hypothesized that the use of SOFA score can diagnose the progress of infection to lung parenchyma (the true definition of VAP) more accurately compared with chest X-ray, the gold-standard diagnosis must be established by more accurate modality such as chest CT, and the accuracy of two diagnostic methods; SOFA vs. chest X-ray, must be evaluated. Or if the authors are willing to demonstrate the use of SOFA score can diagnose gradual VAP more rapidly compared with chest X-ray, they must show the timing of diagnosis of VAP by two diagnostic methods. In the current manuscript, the authors have just compared the SOFA scores between VAP and VAT patients diagnosed by chest X-ray. It is not clear how they want to utilize the SOFA score to distinguish VAP and VAT.

*We fully agree on the importance of clearly describing the purpose of our study.*

*Our study aimed to clarify a practical question for the daily clinical practice, i.e. whether diagnosis of sepsis according to Sepsis-3 criteria, reflecting a greater severity of illness, would be a reliable criterion to early detect or rule out VAP, and therefore decide whether to initiate or not antibiotic treatment in VA-LRTI. In the prospect of a practical diagnostic study, this assessment was made by the time of appearance of clinical signs of VA-LRTI, when radiographical signs of pneumonia on chest X-ray are frequently hidden or delayed*^2–4,7^*.*

*In our study, only chest X-ray was used to distinguish VAT from VAP as CT-scan is not routinely performed for the diagnosis of VAP, due to greater cost, increased time required to obtain images, higher radiation exposure and risks associated with intra-hospital transportation*^2^*.*

*We acknowledge that lung opacities were probably missed in some patients with VAP, given the reported lack of sensitivity of chest X-ray for the detection of pneumonia*^3,4^*. However, the use of chest X-ray allowed us to diagnose VAT and VAP using the same criteria than in the TAVeM study. Accordingly, our definitions of VAP and VAT are consistent with those in which a difference in mortality rates were reported*^5^*.*

*Our results suggest that assessment of severity, especially through sepsis criteria according to the Sepsis-3 definition, exhibit poor performances to early differentiate VAT from VAP. Importantly, we found poor sensitivity and negative predictive value of these criteria to rule out a diagnosis of VAP. As a consequence, these criteria should probably not be used to drive the early initiation of antimicrobial therapy in patients with evidence of VA-LRTI. Especially, the absence of diagnosis of sepsis appears as a bad criterion to decide not to initiate antibiotic treatments.*

*Finally, we agree on the interest of comparing timings of diagnosis of VAP when using both methods, i.e. comparing severity criteria vs chest X-ray. However, our study was not designed to answer this question, but merely aimed to evaluate severity criteria at the time of clinical evidence of VA-LRTI for the diagnosis of VAP, which corresponds to a practical question at the patient’s bedside.*

*All these points have been developed in the discussion and limitations of the revised manuscript. We believe it will help the reader to understand more clearly the purpose of our study, as well as the significance of our results for the daily clinical practice.*

*References:*

1.	Torres A, Niederman MS, Chastre J, Ewig S, Fernandez-Vandellos P, Hanberger H, et al. International ERS/ESICM/ESCMID/ALAT guidelines for the management of hospital-acquired pneumonia and ventilator-associated pneumonia: Guidelines for the management of hospital-acquired pneumonia (HAP)/ventilator-associated pneumonia (VAP) of the European Respiratory Society (ERS), European Society of Intensive Care Medicine (ESICM), European Society of Clinical Microbiology and Infectious Diseases (ESCMID) and Asociación Latinoamericana del Tórax (ALAT). Eur Respir J. 2017 Sep;50(3).

2.	Keane S, Vallecoccia MS, Nseir S, Martin-Loeches I. How Can We Distinguish Ventilator-Associated Tracheobronchitis from Pneumonia? Clin Chest Med. 2018;39(4):785–96.

3.	Butler KL, Sinclair KE, Henderson VJ, McKinney G, Mesidor DA, Katon-Benitez I, et al. The chest radiograph in critically ill surgical patients is inaccurate in predicting ventilator-associated pneumonia. Am Surg. 1999 Sep;65(9):805–9; discussion 809-810.

4.	Graat ME, Choi G, Wolthuis EK, Korevaar JC, Spronk PE, Stoker J, et al. The clinical value of daily routine chest radiographs in a mixed medical-surgical intensive care unit is low. Crit Care Lond Engl. 2006 Feb;10(1):R11.

5.	Martin-Loeches I, Povoa P, Rodríguez A, Curcio D, Suarez D, Mira J-P, et al. Incidence and prognosis of ventilator-associated tracheobronchitis (TAVeM): a multicentre, prospective, observational study. Lancet Respir Med. 2015;3(11):859–68.

6.	Kalil AC, Metersky ML, Klompas M, Muscedere J, Sweeney DA, Palmer LB, et al. Management of Adults With Hospital-acquired and Ventilator-associated Pneumonia: 2016 Clinical Practice Guidelines by the Infectious Diseases Society of America and the American Thoracic Society. Clin Infect Dis Off Publ Infect Dis Soc Am. 2016 Sep 1;63(5):e61–111.

7.	Ramirez P, Lopez-Ferraz C, Gordon M, Gimeno A, Villarreal E, Ruiz J, et al. From starting mechanical ventilation to ventilator-associated pneumonia, choosing the right moment to start antibiotic treatment. Crit Care Lond Engl. 2016 Jun 3;20(1):169.
