# Supplementary material for: Usefulness of Sepsis-3 in diagnosing and predicting mortality of ventilator-associated lower respiratory tract infections
Source: PLoS One. 2021 Jan 14;16(1):e0245552. doi: 10.1371/journal.pone.0245552 (PMC7808583; doi:10.1371/journal.pone.0245552)
Supplement: Attachment — Submitted filename: Response to Reviewers R2.docx [file pone.0245552.s006.docx]

Dear Editor,

Dear Reviewer,

Thank you for allowing us to reply to your comments.

We have provided some additional clarifications which, we believe, have improved the quality of our manuscript.

Please find appended below in italic our response to your concerns:

**Reviewer #3:** Thank you very much for giving me the opportunity to review the revised version of the manuscript entitled “Usefulness of Sepsis-3 in diagnosing and predicting mortality of ventilator-associated lower respiratory tract infections”. I have understood the aim of the study; the authors are willing to evaluate the utility of the Sepsis-3 criteria to diagnosis VAP, because chest X-ray lacks adequate sensitivity or specificity to diagnose pneumonia. However, according to the fact, the VAP diagnosis by chest X-ray in this study might not be accurate, and the diagnostic utility of Sepsis-3 criteria evaluated based on the inaccurate VAP diagnosis has no meaning. To prove the authors’ hypothesis, the gold-standard diagnosis of the VAP must be performed by more accurate modalities such as chest CT. The design and data set in this study cannot solve the question raised by the authors. It is necessary to comprehensively reconsider the study aim and design.

*We would like to thank you for this comment. As you are pointing out, our study evaluated the value of the Sepsis-3 criteria for the diagnosis of VAP, as the interpretation of the chest X-ray can be difficult in daily practice.*

*We fully understand your question regarding the relevance of the radiographic criteria in our study. Nevertheless, it should be remembered that the diagnosis of VAP was made with full knowledge of the patient's medical record. This included the possibility of observing the appearance of prior opacities on the chest X-ray, or secondarily unmasking a differential diagnosis of VAP, such as cardiac overload, atelectasis, or pleural effusion. In these different cases, the diagnosis of VAP could reasonably be made ultimately on the basis of the chest x-ray, while still being difficult in daily practice at the patient's bedside. Therefore, it seems to us that chest X-ray, which was used in the largest international study describing the characteristics of VAT and VAP^[1]^, was relevant in our study.*

*On the other hand, CT scan was only used in a minority of cases to diagnose VAP in our patients, and therefore could not be used in our study. This situation reflects the low practical use of this tool to diagnose VAP. Thus, although we recognize the potential value of CT scan in a comprehensive and mechanistic study, its practical use for the diagnosis of VAP is likely to be limited.*

*We are aware of the importance of these issues in the interpretation of our study. Accordingly, these different points have been developed in the limitations section of our manuscript.*

*We hope that these explanations have helped to remove your concerns about the value of this study, and remain at your disposal to answer any further questions you may have.*

1. Ignacio Martin-Loeches and others, ‘Incidence and Prognosis of Ventilator-Associated Tracheobronchitis (TAVeM): A Multicentre, Prospective, Observational Study’, *The Lancet. Respiratory Medicine*, 3.11 (2015), 859–68 <https://doi.org/10.1016/S2213-2600(15)00326-4>.
